# Scientist Citizen: An Interview with Bruce Alberts

**DOI:** 10.1371/journal.pgen.1002743

**Published:** 2012-05-31

**Authors:** Jane Gitschier

**Affiliations:** Departments of Medicine and Pediatrics and Institute for Human Genetics, University of California San Francisco, San Francisco, California, United States of America

As the *PLoS Genetics* editorial board prepared for its first scientific meeting in San Francisco, its leaders proposed that I conduct an interview “live” as a feature of the proceedings, and I knew immediately whom I wanted to snare: Bruce Alberts, our own UCSF (University of California San Francisco) faculty member extraordinaire, who had long been on my radar screen for an interview.

**Image 1:**
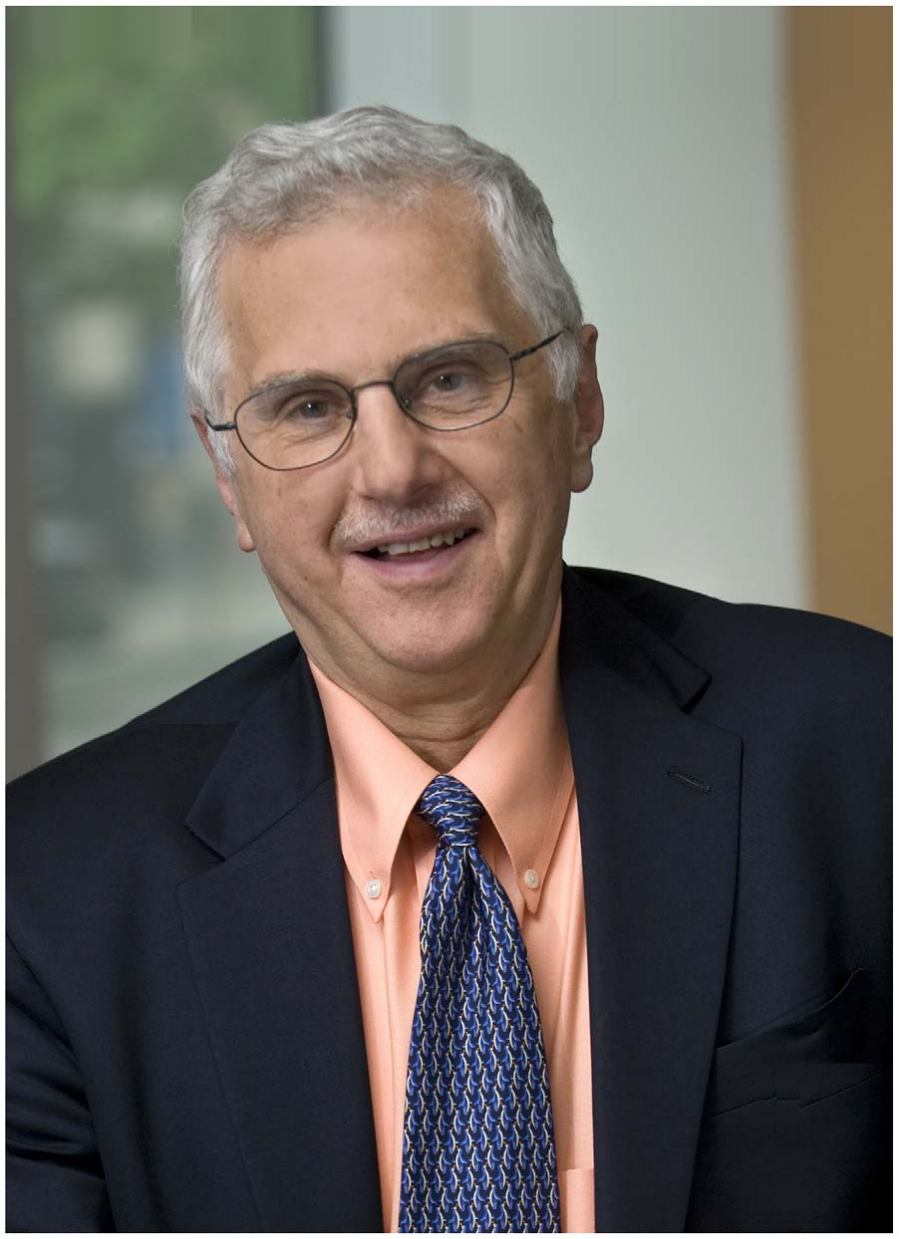
Bruce Alberts. Photograph by Tom Kochel, courtesy of the Department of Biochemistry and Biophysics, The University of California San Francisco.

Alberts (Image 1) is not only a remarkable scientist, he is also one of our country's most passionate voices for science education. Indeed, his astonishing record of contributions to our understanding of DNA replication and molecular machines is now almost eclipsed by his public service. To consider what Alberts has already accomplished in his lifetime is humbling: one of the founding co-authors of the highly successful textbook *Molecular Biology of the Cell*, originator of the UCSF Science and Health Education Partnership with the San Francisco public schools, and president of the National Academy of Sciences (NAS) for 12 years. Yet, at age 74 he is still going strong: I counted 56 committees on which he currently serves; he recently completed his tour of duty with the US Department of State as a science envoy to Pakistan and Indonesia; and he steers the journal *Science* as its editor-in-chief.

Clearly, Bruce seems to have trouble saying “no”, and, as head of the Bay Area Science Festival, which coincided with our board meeting, he also happened to be in town and worked us into his schedule. At the appointed hour, I met Bruce on a drizzly November afternoon at the Hyatt at Fisherman's Wharf. He arrived in his silver Honda Civic, its bumper emblazoned with an NAS sticker, and claimed to be a bit befuddled by a lack of coffee, which we immediately supplied, and the absence of his hearing aid, which we didn't. But he brought with him the curiosity, goodwill, energy, humility, and delight that infuses everything he does and makes him so effective.

**Gitschier:** Over the course of your life, you've written so much about public policy and education, and you've been involved in a lot of important decisions, but I want to start way back at the beginning—becoming a scientist.

**Alberts:** I grew up in the suburbs of Chicago in a town called Glencoe on the north shore. I went to a high school called New Trier, and I've always been disorganized, so I was late for everything. I signed up for biology so late I couldn't get into the class, so I never took any biology until college.

So instead I took amateur radio. I learned how radios work and got an amateur license and it was, I'm sure, a better experience than biology in a memorize-all-the-words class.

**Gitschier:** So you were one of those guys up late at night talking to people in Siberia?

**Alberts:** Yes, mostly Morse code.

**Gitschier:** Morse code? So, was either of your parents a scientist?

**Alberts:** My father was a mechanical engineering undergraduate and then worked in the patent office in Washington and went to night school and became a patent lawyer. He set up his own patent practice in Chicago. His first dream was that I would take over his business, but that would be—even if I liked it—very stultifying, working for your father. His second dream was that I would become a doctor so I could help take care of him; so he wasn't that disappointed when I went to Harvard as a premed.

At that time, my friends and I had no idea you could actually be a scientist. We knew Albert Einstein had worked in a patent office, and I guess I had an image that there was no such thing as a scientist that got paid. When I went to career night to see what I could do with chemistry, because that was my favorite class in high school, there were only two choices that the parents who spoke presented. One was a chemical engineer, and that seemed really boring: it was huge tanks with pipes; I still remember the slides. The other was this doctor who tried to convince us that chemistry and science are embedded in medicine, which I don't think it really is for most doctors.

So I went to college as a premed, and you have to take all these science courses. And I was in these laboratory courses three afternoons a week for three years, which I hated. They are so unlike science, basically just cooking. In my third year, the physical chemistry lab was the worst of all. So I asked whether there was any way I could take the physical chemistry course and drop the lab.

In this way, I discovered accidentally that I could work in a research lab. I had a tutor whose name is Jacques Fresco, who became a professor later at Princeton University, but he was then a post-doc with Paul Doty, a very famous, distinguished chemist. He had something like 37 people in his lab, and I really was working with Jacques Fresco. I fell into a wonderful problem that Jacques had set up for my senior thesis. We published one paper in *PNAS*, another one in *Nature*. So I thought science was very simple, but that was very misleading!

The first thing I was planning to do in graduate school was to solve the genetic code!

**Gitschier:** OK! What year are we talking about, Bruce?

**Alberts:** This was 1960. I wrote a senior paper for a course where I proposed these experiments. Actually there's a man people probably don't remember anymore—Ernst Freese. He had done all these beautiful mutagenesis studies in T4 bacteriophage, and described hot spots for mutation, I think it was the RII locus, showing that you can map down to the individual nucleotides of the sites by recombinational analysis. He showed that different mutagens had different hot spots; they all created mutations in different places. This was way before the famous poly-U experiment [of Marshall Nirenberg].

So my way of solving the genetic code was to figure out what [nucleotide sequences] those chemicals preferred by taking advantage of a technique that Arthur Kornberg had recently published—nearest neighbor analysis—where you take DNA polymerase and incorporate one ^32^P-labeled nucleotide at a time. And of course that [label] goes in the 5′ phosphate. And then you hydrolyze with a set of nucleases that makes 3′ phosphates …

So I had this whole scheme. I got an A on this paper, so I thought I might as well *do* this. And I had no idea about research strategy. Paul Doty told me I had been so successful, he wanted me to stay there as a graduate student, and then he said I could work on whatever I wanted to, so I tried to make that my PhD thesis.

Well, first of all, you couldn't buy ^32^P triphosphate so I had to go to Oak Ridge [Tennessee] for three months to learn how to make triphosphates. I must be heavily irradiated because every two weeks I'd grow 60 mCi of ^32^P-fed *E. coli*! I spent two years doing these nearest-neighbor experiments, and finally I realized it wasn't going to work. And why …

At that point Francis Crick came to visit Paul Doty, and so Doty prodded us—one graduate student at a time—to explain what we were doing to Francis Crick. So I told him my research plan. I didn't tell him my results. And the first thing he said was, “Have you done the control? Can you completely hydrolyze DNA after it's been treated with these agents to the 3′ phosphates?” And, of course, I had just discovered you couldn't! Basically I didn't know enough science to do the control first. That was my first lesson in research strategy.

Then, I decided I was going to solve how double-stranded DNA replicated. A very ambitious, crazy PhD thesis project. Nobody in the lab was working on this, just me. At the time everyone thought that DNA was replicated by one enzyme: DNA polymerase. So I had all these crazy theories about why it wouldn't work on double-stranded DNA under the conditions that Arthur Kornberg had used. And again, I made another model, and I wrote a graduate student paper with all these theories in it. Jim Watson read it and liked it. I was totally overconfident. So I spent the next three years trying to test my theories. Of course, every time I did a test, it came out “no”: there was no indication that my theory was right.

**Gitschier:** So you went through a series of theories?

**Alberts:** Failures! So eventually I decided to do my thesis on something I observed while trying to test out my theories. The DNA in *E. coli* is naturally cross-linked; it's true in *all* organisms. And I even did this on calf thymus.

So after five years I was going to graduate, and there was this formality of an oral thesis committee meeting. And nobody had ever failed this, as far as anybody knew. I had already given up my apartment. I had my wife and one-year-old baby. I had tickets to go on my post-doc in Geneva in two weeks. And I walk into the room and there's Matt Meselson and a bunch of famous people … Wally Gilbert and so on … and the first thing they tell me, before I even say anything, is that they didn't believe my thesis and that I wasn't going to get my PhD.

**Gitschier:** So they read your thesis draft and said, “Forget it.”

**Alberts:** Yeah. They didn't believe what I had found and gave me all these additional experiments. Just like referees [for journal publications]! So the first month I spent analyzing my failures, and wondering whether I should quit. Should I even be a scientist?

And that was a very important learning experience for me. I had decided that experimental strategy was *everything* in science, and nobody had ever told me anything about this. Anyway, six months later I had finished all those experiments, and they confirmed what I had originally said.

**Gitschier:** How did you get from DNA replication to cross-linking?

**Alberts:** We thought the *E. coli* chromosome was linear. So my theory about DNA replication involved a whole complicated way of starting at the end of the linear molecule, which would form a hairpin helix and be detectable as a cross-link between strands. You'd denature DNA in high dilution, and cross-linked molecules would renature. I was mapping all those sites, and trying to see if they were at the origin of replication, which they weren't: they were elsewhere!

But we didn't have any idea that replication was carried out by a protein machine involving a whole complex of proteins. And then, by luck, I had arranged to go to Geneva for a post-doc. The whole idea was to work on your own project. It was much simpler in those days.

I had gone to work with Alfred Tissières, officially, but I ran into Dick Epstein. He had published a Cold Spring Harbor Symposium paper in 1963 about all the mutational analysis he, with Bob Edgar and others, had done at Caltech categorizing temperature-sensitive and amber mutations in T4 bacteriophage. And they had shown very clearly—and this was right in the middle of my thesis and if I had read it, I would have stopped doing those experiments—that there were at least seven different proteins needed for replication of T4 DNA. *One* of them was DNA polymerase.

I suddenly realized that my whole hypothesis, and the way everybody was thinking about DNA replication, as far as anyone I knew, was totally misguided. That made me change to work on the T4 system.

**Gitschier:** So your first paper on T4 came out in …

**Alberts:** 1968 was the gene 32 protein.

**Gitschier:** Okay.

**Alberts:** Anyway, I skipped something: Part of my conclusion from studying my failures—and I was going to incorporate it in my post-doc—was to never do an experiment where either result you get doesn't mean something, because all those thesis experiments I did meant nothing because my theory was wrong and nobody cared.

And the second conclusion was to try and do something different from other people because I had been in a lab where they were racing [Marshall] Nirenberg and [Har Gobind] Khorana to solve the genetic code. I thought that this is no way to spend a scientific career. Even if they beat them by two weeks, what was the point?

So I decided that I wanted to do something different. One way to do that is to develop a new method that then allows you to do something that other people can't do. By the time I got to Geneva, I decided I was going to try to develop a DNA column that would allow me to purify proteins that bound to DNA. I spent a lot of time doing fancy things—chemical linkages—and by accident, just by being sloppy—I let a sample dry—I found that if you dry DNA on pure cellulose with no chemical derivatives, it sticks very well. Both single-strand and double-strand DNA. That was my first independent paper.

**Gitschier:** So you wrote a methods paper.

**Alberts:** No, it *was* a methods paper, but of course you had to have a *result*, because otherwise you can't get it published.

**Gitschier:** Well, I'm bringing this up because we [the *PLoS Genetics* board] just had a big discussion before you showed up about publishing methods papers.

**Alberts:** We disrespect methods, which is, I think, a mistake because they are so important.

Anyway I had used T4, I infected with all the different mutants, and I found that for the gene 32 mutant, a huge band that normally bound to single-stranded DNA, was missing. And so I purified that protein, starting with DNA cellulose, followed by a few more steps and showed that it was the first single-stranded binding protein. That was part of the 1968 *Nature* paper.

Princeton had insisted I come right at the end of my first postdoctoral year. I had a job as an assistant professor there before I left. This was a completely different era; molecular biology was expanding. All Paul's students had jobs even before they graduated!

**Gitschier:** Wow. So, then you had to start to purify all these protein components.

**Alberts:** Yeah. And then in 1976, one day we added all seven proteins to the double-stranded DNA and we had the right magnesium concentration: we could make DNA. That was another great day in my scientific career because I had been trying to do this since 1960. And failing. But it took us another six years or so to figure out what those proteins did, because when you mix six or seven things together and you have a reaction, you don't know what's going on.

I think we have greatly underestimated the need for this kind of protein biochemistry: reconstituting systems. We need to get many more young people doing this kind of work. It doesn't go quickly, and you don't get a lot of publications quickly, so I worry that our current system is pressuring people to do science that is not as effective as it should be.

**Gitschier:** You've just described two big days of scientific excitement. Are there any others you want to talk about?

**Alberts:** One I still remember was coming to a realization that the DNA synthesis on the two strands is coupled. Because we never had thought of that. There's a leading strand—the DNA polymerase is going like this [gesturing with hands], and the lagging strand is going backwards and making the Okazaki fragments.

I was preparing for a seminar somewhere—and this is why giving seminars makes sense—and I was scribbling around and it led me to something called the “trombone model”, which we subsequently tested and found was correct. In fact, the leading and lagging strand DNA polymerases are tied together, and there's this loop that comes out back.

That changed my whole conception about how *life* works, personally. I mean we knew about complexes, like ribosomes, but this is a different kind of protein machine because it's assembled during the reaction. It is not a permanent complex, and like all machines, there's an ordered, directional movement caused by coupling to an energy source, usually ATP hydrolysis.

Again, I recognized a lot of these things from writing a textbook, *Molecular Biology of the Cell*, because it forces you to read and to think. I wrote the protein chapter, and I suddenly realized that this ATP hydrolysis by proteins, driving them through conformations, was a critical element of life. Unidirectional conformation changes, driven by bound nucleotides that are hydrolyzed—just *everywhere*!

**Gitschier:** Going back to when you were a graduate student, you said you took a month and thought about your failure. Actually it wasn't really a failure.

**Alberts:** Well, I failed my thesis. We've all made mistakes. Students love hearing about all my failures, the highlight of my talks. Then I emphasize how we all learn from failures and why old people are useful, because we fail so many times and learn a lot.

I've observed you can't make a good graduate student into a successful scientist. They have to make *themselves* into successful scientists. To be a good mentor you have to help them, but not so much that you don't allow them to have that chance to make their own discoveries. Only that way do they get enough self-confidence to fight their way through the difficulties that any project will have.

One of the things I think is very important for students and for every scientist is to take a day every couple weeks and just sit there with a blank piece of paper and start writing down what you're doing. With the clear attitude in mind—which is *always* true—that there is a better way to do it. I mean if you believe that, then you work harder.

Also, you realize that people are good at different things and there's no *one* kind of intelligence. And this is part of why I've been working a lot in the last 25 years on science education and education in general. We have to enable kids to recognize that their job in education is to discover what they are good at and what they enjoy, with the assumption that everybody is really different and they *are* going to be good at something. So many kids get turned off of school now because there is only one way to succeed. This is a long conversation, but I've been writing a lot about the importance of science education and the need to redefine what we mean by science education.

**Gitschier:** OK, I want to jump to what seems to be a pivotal time for you. In 1985 you wrote a *Cell* perspective about small science, and then the next year you were asked to head the National Academy of Sciences panel about the human genome project.

**Alberts:** Right.

**Gitschier:** Was there a correlation between writing that piece and then being asked to chair the committee?

**Alberts:** Yes. I didn't realize it at the time. It's hard to remember this now, but most biologists were strongly against sequencing the human genome, because at that time it was going to take 10,000 person years and it was going to look like big physics.

So there was a committee set up by the Academy. It had Jim Watson, Sydney Brenner, Dan Nathans—they were strongly in favor of it. And then there were people on the other side. David Botstein and Shirley Tilghman, now famous but then young scientists, who were talking against it. So this was an incredible committee and they were looking for a chair. And they called me, just out of the blue. I had never even thought about this problem. And they said they wanted somebody who had never even thought of the problem.

But what they really wanted, I think, was the fact that I had been encouraging small science in biology. And that if I were to chair, it would show that the committee wasn't biased to big science.

We actually got everybody to agree, even the opponents, that there should be a genome project, but we really re-defined it. Don't sequence a lot of DNA until you get the cost below 50 cents per base pair, and do a lot of the practicing on yeast and *E. coli* and model organisms, because there'll be homologies and this will allow you to both develop the technologies while you're preparing these technologies and interpret the human genome. And of course, we didn't realize how true that was going to be.

**Gitschier:** How did you get the committee come to a consensus?

**Alberts:** So, this is an art form, getting a committee to reach consensus.

**Gitschier:** And you must be a master of this by now.

**Alberts:** The staff officer John Burris and I worked very hard on a strategy that would allow a consensus to be built. We knew that if we started making recommendations right at the beginning, everybody would be arguing, there would be no point. And so, we designed a strategy where we were not going to discuss any of the controversial issues until we had educated ourselves.

So we invited scientists who were on the front lines actually doing this stuff to come in, to figure out how hard it would be to sequence the human genome and what the problems were. We heard from people like Maynard Olson that this was not going to be simple. Some of the senior scientists viewed this the way I had viewed solving the genetic code—on paper you could make arguments about why it would be easy, but there were many complications.

By the time we had written the preliminary chapters about mapping and all the background information we could already agree on, intellectual property and things like this, then we asked—almost at the last meeting—whether it should be a project. And by that time we could all agree.

That was the first policy work I had ever accomplished, so then they thought I could do anything, and the Academy kept putting me on other committees and putting me in charge of stuff. And that led to my change in career eventually.

**Gitschier:** Because quite soon after that actually you moved to the National Academy, right?

**Alberts:** Well, the report came out in 1988, and then in early 1992, they set up a committee to look for the next president of the Academy. They called me, and I said I didn't want to do it. They realized that I didn't want to do it, but the way they got me to do it was saying that I was already working a lot on science education in San Francisco. And they said that I could use the Academy as a platform to work on advancing science education. The Academy was in the middle—and I was on the committee—of preparing the first ever National Science Education Standards for the United States.

So, at any rate, there was a lot of distressing back and forth and agonizing on my part. Because it's a full-time job, there was no way I could run my lab in San Francisco and be in Washington full-time. So I had to close down my lab.

**Gitschier:** So, tell me about that. Was that …

**Alberts:** Painful! So what I did was I just didn't take anybody else, and as soon as my lab got down to the last three people, they went to other labs, and that was it. My lab closed in 1995.

**Gitschier:** And then, you must have liked the job well enough, because you signed up for a second six years.

**Alberts:** That was surprising! Actually, I had told them I would do it for only four years, but by the time four years was over, my lab was closed, and I didn't know what else I would do!

**Audience Question:** Regarding your transition from laboratory to one of service: how did your institution support that transition? It's a bold decision that not many would take.

**Alberts:** A lot of people said the same thing.

You look at the current political system in the US. It's incredibly depressing. These kinds of statements that “scientists only believe in climate change so that they can get a grant.” This kind of stuff couldn't be said if we actually had a population that understood what science is. We have a fantastic scientific community, and if we don't unleash them and give them credit for working on these things, then I don't think our country is going to prosper.

Every ten years the Academy publishes a booklet called “Science, Evolution and Creationism” [available online], and before the last one in 2008, the Academy hired one of the companies that put people behind a one-way mirror and interview them to see what they think about some new product. But this question was, “How do they think about science and creationism?”

And the staggering message from these college-educated adults is that they don't see any difference between science as a belief system and religion as a belief system. So basically, the preacher tells them what religious people believe, the scientists tell them what scientists believe, and [they think] “I can choose either one.” And the reason they can say that is that they don't understand what we call “science as a way of knowing”. That it is not a *belief* system, that it is an evidence-based community process.

This is just unbelievable, that our American public can determine our future without understanding the fundamental issues about scientific facts. If the population isn't prepared to deal with these kinds of issues, to think rationally and respect evidence, then I think the country is really in danger.

There is a movement called the Young Academy Movement. It started in Germany maybe 12 years ago—where the senior academy picked out 80 or 100 35-year-old scientists across all fields, and they are given a license for five years to do something for their country and are given a few resources. Then it spread to the Netherlands and now there are about ten of them throughout the world. There is an organization the Germans are supporting called the Global Young Academy to help other countries set up academies.

Wouldn't it be great if we had in the US a hundred young scientists who are gifted in talking to the public and who are interested in doing this? We have to do something! We are dying here. Until we create that kind of connection between the scientific community and other parts of our society, we're not going to get science imbued into these areas. And the idea is so horrible—that a faculty member is valued by how much grant money and overhead they bring to a university—this is a disaster. I'm very passionate, and the question is a good one. We need to value those things!

